# Prenatal wellbeing of mothers, their partners, and couples: a cross-sectional descriptive study

**DOI:** 10.1186/s12884-023-05790-4

**Published:** 2023-06-22

**Authors:** Tia Mäkelä, Terhi Saisto, Katariina Salmela-Aro, Jenny Miettinen, Harri Sintonen, Hanna Rouhe

**Affiliations:** 1 Department of Obstetrics and Gynecology, Helsinki University Hospital, PO BOX 140, Helsinki, 00029 HUS Finland; 2 University of Helsinki, PO BOX 4, Helsinki, 00014 Finland; 3 Department of Educational Sciences, University of Helsinki, PO BOX 9, Helsinki, 00014 Finland; 4 Espoo Health Care Center, City of Espoo, PO BOX 1, Espoo, 02070 Finland; 5 Department of Public Health, University of Helsinki, PO BOX 20, Helsinki, 00014 Finland

**Keywords:** Posttraumatic stress disorder (PTSD), Fear of childbirth, Depression, Health-related quality of life (HRQoL), Prenatal care

## Abstract

**Background:**

Prenatal posttraumatic stress symptoms (PTSS), fear of childbirth (FOC), and depressive symptoms have been related to various negative effects during pregnancy, childbirth, and in the postnatal period. This study evaluates the prevalence of PTSS, FOC, depressive symptoms, and health-related quality of life (HRQoL) among pregnant women, their partners, and as couples.

**Methods:**

In a cohort of 3853 volunteered, unselected women at the mean of 17th weeks of pregnancy with 3020 partners, PTSS was evaluated by Impact of Event Scale (IES), FOC by Wijma Delivery Expectancy Questionnaire (W-DEQ-A), depressive symptoms by Edinburgh Postnatal Depression Scale (EPDS), and HRQoL by 15D instrument.

**Results:**

PTSS (IES score ≥ 33) was identified among 20.2% of the women, 13.4% of the partners, and 3.4% of the couples. Altogether, 5.9% of the women, but only 0.3% of the partners, and 0.04% of the couples experienced symptoms suggestive of phobic FOC (W-DEQ A ≥ 100). Respectively, 7.6% of the women, 1.8% of the partners, and 0.4% of the couples reported depressive symptoms (EPDS ≥ 13). Nulliparous women and partners without previous children experienced FOC more often than those with previous children, but there was no difference in PTSS, depressive symptoms, or HRQoL. Women’s mean 15D score was lower than partners’ and that of age- and gender-standardized general population, while partners’ mean 15D score was higher than that of age- and gender-standardized general population. Women whose partners reported PTSS, phobic FOC, or depressive symptoms, often had the same symptoms (22.3%, 14.3%, and 20.4%, respectively).

**Conclusions:**

PTSS were common in both women and partners, as well as in couples. FOC and depressive symptoms were common in women but uncommon in partners, thus they rarely occurred simultaneously in couples. However, special attention should be paid to a pregnant woman whose partner experiences any of these symptoms.

## Background

Mental stress and disorders are often unrecognized in maternity care [1–3]. Recognition, further support, and admission to relevant care aim at better well-being in families with infants. The most common mental disorder during pregnancy is depression with a wide prevalence estimation of 5–67% in women [4–6] and 4–32% in partners [4]. Prenatal depression has been related to various adverse effects during pregnancy, childbirth, and the postnatal period both in women and their partners [1, 5, 7, 8] (such as increased risk for preterm birth [1, 5], low birthweight [1, 5], caesarean section  [5], maladjustment to parenthood [8], poor mental health [7], and developmental and mental problems of the child [1, 8]). There is evidence of in-between couples’ depressive symptoms [4, 8, 9].

In women, fear of childbirth (FOC) is associated with depressive symptoms [10] and seems quite common during pregnancy with 4–31% prevalence in earlier studies [11]. The term FOC is used as a broad label for many kinds of anxieties and fears that a person can experience in relation to childbirth [11]. Pregnant women have more FOC [12] than their partners as the partners’ prevalence of FOC is 0–14% [12, 13]. FOC in couples is poorly studied. Both in women and partners, FOC has been related to various adverse effects, such as poor mental health [10, 13, 14], dissatisfaction with the relationship [14], increased (emergency) caesarean section rates [12, 15, 16], negative childbirth experience [15, 17, 18], and viewing or experiencing parenthood as more difficult [13, 18]. Moreover, FOC is one of the major predisposing factors for PTSS or PTSD after childbirth [19]. On the other hand, PTSD after childbirth often leads to fear of childbirth in the next pregnancy and can lead to a reduced desire for having more children or a request for a caesarean sections.  [19, 20].

It might be that posttraumatic stress disorder (PTSD) is also quite a common mental disorder during pregnancy as its prevalence varies largely from 0 to 40% [21–23]. PTSD diagnosis requires trauma (the person experienced, witnessed, or confronted a situation(s) of an exceptionally threatening or catastrophic nature) and four sets of symptom clusters (intrusion or re-experiencing; avoidance; negative alterations in mood or cognitions, and increased arousal) [24]. Symptoms must have lasted for at least one month and must significantly impair functioning [24]. The term posttraumatic stress symptoms (PTSS) is used when symptoms do not quite fulfill all the PTSD criteria, or when self-report measures are used to screen for PTSS/PTSD. Perinatal PTSD is known to co-occur with other psychiatric disorders, such as depression, and has negative effects on the individual, the relationship with partner and child, pregnancy, and childbirth [21–23, 25]. Prenatal PTSD is less studied, but it might be a risk factor at least for abnormal fetal growth, miscarriage, hyperemesis, preterm contractions, and preterm birth [22, 25]. While PTSD and PTSS during pregnancy have been studied among pregnant women [21–23, 25], hardly any studies have been conducted on either among partners’ or woman’s and her partner’s (couple’s) shared PTSS or PTSD.

Health-related quality of life (HRQoL) is a concept consisting of an individual’s perceived physical and mental health [26]. Pregnant women are known to have worse self-rated health than their partners [27] and worse HRQoL than the general female population [26]. It is still unclear which domains of life are impaired during pregnancy and whether HRQoL changes in partners during pregnancy.

In earlier studies, there are high variations in the prevalence of depression, FOC, and PTSD in women during pregnancy. Moreover, hardly any or only a few studies have been conducted on the prevalence of prenatal PTSD and FOC in partners or couples, although there is evidence of in-between couples’ depressive symptoms [4, 9]. To get a better understanding of the mental well-being of our unselected pregnant population in Finland, our study object was to evaluate and compare the prevalence of depressive symptoms, FOC, and PTSS/PTSD, as well as HRQoL, among pregnant women, their partners, and as couples. Moreover, we aimed to examine if there were differences in the prevalence of these conditions and in HRQoL between those without and with previous children. We were also interested in how well the Visual Analogue Scale (VAS) worked in identifying fear of childbirth among women and partners.

## Materials and methods

### Participants

An unselected population of Finnish- or Swedish-speaking pregnant women and their partners attending the routine first trimester ultrasound screening in maternal clinics in Helsinki and Uusimaa Hospital district between April 2014 and August 2015 were offered a chance to participate in this cross-sectional study. Altogether, 3853 pregnant women and 3020 partners of which 3020 couples where both had replied - completed the survey. Women and partners were asked to return the surveys separately by post or when they came to the second ultrasound screening at gestational age 19–21 weeks. They completed the surveys on average at gestational age 16 + 5 weeks (standard deviation, SD 3.9, range 5–40). This study has been approved by the Helsinki University Hospital and by the local ethics committee (Helsinki and Uusimaa Hospital District Ethical Committee for women, children, and psychiatry) (approval number 250/13/03/03/2013). All participants gave written informed consent.

### Questionnaires

In the surveys, pregnant women and their partners were asked to report their background information (Table 1) and to answer questionnaires concerning PTSS, FOC, depression, and HRQoL descripted in more detail below.

To measure PTSS and PTSD, we used the Impact of Event Scale (IES) revised version (IES-R), which was developed to assess subjective distress caused by any previous traumatic events [28]. The pregnant women and their partners were asked to identify a specific stressful life event (open question but examples of death or sickness of loved one, divorce, accidents, experiences related to own health, or unemployment were given), to specify timing of this event, and then to indicate how much it distressed or bothered them during the past seven days. The IES-R contains 22 statements about intrusion (intrusive though/feelings/imagery, nightmares, dissociative-like re-experiencing), avoidance (decreased responsiveness, avoidance of feelings, situations, and ideas), and hyperarousal (irritability, hypervigilance, difficulty concentrating, other emotional disturbances) rated on a Likert scale from 0 (not at all) to 4 (extremely). The total score ranges from 0 to 88 and higher scores indicate more distress. The Cronbach’s α was 0.95 both in women and partners. Scores of 33 or over signify the likely presence of PTSD [29]. Those with scores that exceed 24, who do not have full PTSD, have either partial PTSD or PTSS [30]. The IES sum scores could be counted in 3268 (84.8%) women, 2408 (80.1%) partners, and 2119 (70.2%) couples.

To measure FOC, we used the Wijma Delivery Expectancy Questionnaire (W-DEQ-A) [31] and VAS [32]. The W-DEQ-A is a standardized screening method to measure FOC in women [31]. It has also been used for partners [13, 12]. The W-DEQ-A scale contains 33 statements about childbirth rated on a Likert scale from 0 (not at all) to 5 (extremely). Total scores range from 0 to 165, with higher scores indicating higher FOC. The original W-DEQ-A is in English and Swedish and was translated into Finnish in a previous Finnish study after approval from the copyright holder [32]. The Cronbach’s α of the W-DEQ-A was 0.94 in women and 0.91 in partners. A W-DEQ-A sum score of ≥ 85 has been used to suggest severe FOC [1] and a score of ≥ 100 to suggest phobic FOC [4, 16]. The W-DEQ-A could be counted in 3583 (93.0%) women, 2767 (91.6%) partners and 2596 (86.0%) couples. After filling in the W-DEQ-A, the participants were asked to mark an” X” on a 100-millimeter VAS line. The VAS scale ranges from 0 (feeling confident about childbirth) to 100mm (feeling extremely afraid of childbirth). VAS score ≥ 50mm has been considered to suggest severe FOC [32]. The mark in the VAS line was readable in 3818 (99.1%) women, 2985 (98.8%) partners, and 2963 (98.1%) couples. Both VAS score and W-DEQ-A score were available in 3564 (92.5%) women and in 2751 (91.1%) partners.

To measure depressive symptoms during pregnancy, we used the Edinburgh Postnatal Depression Scale (EPDS). The EPDS has been developed to assess postnatal depressive symptoms in women [33], but it has also been validated for partners [34]. Nowadays, it is also commonly used to measure depressive symptoms during pregnancy both in women and partners [4–7, 35]. The EPDS contains 10 statements about mood and feelings rated on a Likert scale from 0 to 3. Total score ranges from 0 to 30, and higher scores indicate more depressive symptoms, which may be related to depression. The Cronbach’s α was 0.84 in women and 0.71 in partners. The EPDS sum score of ≥ 10 has been used to screen people suffering from mild or possible depression and the sum score of ≥ 13 to screen people with probable clinical depression [4, 6, 7, 35]. The EPDS could be counted in 3775 (98.0%) women, 2956 (98.3%) partners, and 2897 (95.9%) couples.

To measure HRQoL we used the 15D instrument (15D). The 15D is a generic, fifteen-dimensional, standardized, self-administered instrument that can be used both as a profile and a single index score measure [36]. The health state descriptive questionnaire is composed of the following dimensions: mobility, vision, hearing, breathing, sleeping, eating, speech (communication), excretion, usual activities, mental function, discomfort and symptoms, depressive symptoms, distress, vitality, and sexual activity. For each dimension, the respondent chooses one of the five levels best describing his/her state of health at present. A set of population-based preference or utility weights is used to generate the dimension level values and the 15D score (single index number) on a 0–1 scale [36]. The Cronbach’s α of the 15D score was 0.77 in women and 0.79 in partners. The 15D score could be counted in 3715 (96.4%) women and 2914 (96.5%) partners. An earlier study estimated the minimum important change in the 15D scores and reported a change or difference of ± 0.015 as clinically important [37]. There is no universal cut-off value for good HRQoL in the 15D, as HRQoL is a highly age-dependent variable. The HRQoL results of the pregnant women and their partners were compared with each other and with those of representative samples of the general population from a previous study [38] with same gender, weighted to reflect the age distribution of the women and their partners.

### Statistical analyses

Statistical analyses were carried out using IBM SPSS Version 21.0 statistical software package (Chicago, USA). Descriptive statistics are reported as means, standard deviations (SD) and ranges of the scores. We also investigated how many pregnant women (all, nulliparous, parous), partners (all, with and without previous children), and couples together exceeded the cut-off values found in earlier studies. Three different comparisons were made for PTSS, FOC, and depression with Mann-Whitney U-tests for abnormally distributed variables, independent samples t-tests for normally/normally alike distributed variables, and Chi-Square tests for categorical variables. The first comparison was between women and their partners, the second between nulliparous and parous women, and the third between partners without and with previous children. Independent samples t-test was used to test the statistical significance of the differences in the mean scores of HRQoL separately between pregnant women and partners and age-standardized, same-gender general population from a previous study [38]. A probability level of ≤ 0.05 was considered statistically significant. All possible data were included in the analyses. If there were missing values, those respondents were excluded from that index analysis. To examine how well the VAS score worked in identifying fear of childbirth among women and partners, sensitivity and specificity in relation to W-DEQ-A were calculated.

## Results

56.2% of the women were nulliparous and 43.8% parous. Partners’ parity was reported in 76.2%, and of those 43.1% had no previous children and 56.9% had previous children. The women were a bit younger than their partners (Table 1). According to the screening tools, women reported more symptoms associated with PTSS, FOC, and depression than their partners, when compared with means and known cut-off values (Table 2, p < 0.001 in all comparisons). Altogether, 199 women (5.2%) and 2 partners (0.07%) had both VAS score ≥ 50 mm and W-DEQ-A ≥ 100. With same cut-off values the VAS score’s sensitivity was 94.3% and specificity 63.1% in women, and respectively 28.6% and 85.4% in partners.

**Table 1:** Background characteristics of the study population

Background characteristics	All women	Nulliparous	Parous	All partners	Partners without previous children	Partners with previous children
Age (years), mean ± SD (range)	31.2 ± 4.6 (17–48)	30.2 ± 4.6 (17–46)	32.5 ± 4.3 (20–48)	32.9 ± 5.2 (17–59)	31.1 ± 4.5 (17–49)	34.6 ± 5.3 (20–59)
Twin pregnancy, n (%)	54 (1.5)	32 (1.5)	22 (1.0)	NA	NA	NA
Cohabitation, n (%)	3664 (95.4)	2021 (94.2)	1629 (97.1)	2920 (97.4)	956 (97.2)	1277 (98.1)
Academic degree education, n (%)	1387 (36.2)	781 (36.4)	597 (35.7)	956 (32.0)	344 (34.9)	411 (31.8)
Full-time job, n (%)	2961 (77.9)	1877 (88.3)	1070 (64.5)	2702 (90.2)	870 (88.3)	1180 (90.9)
Native country Finland, n (%)	3676 (95.7)	2052 (95.6)	1608 (95.9)	2807 (93.3)	924 (93.2)	1218 (93.5)
Smoking during pregnancy, n (%)	483 (12.9)	314 (15.0)	167 (10.3)	NA	NA	NA
Quit smoking during pregnancy, n (% of smokers)	378 (78.3)	259 (82.5)	117 (70.1)	NA	NA	NA
Previous miscarriages, n (%)	835 (21.7)	339 (15.7)	492 (29.3)	NA	NA	NA
Previous terminations of pregnancies, n (%)	444 (11.5)	217 (10.1)	224 (13.3)	NA	NA	NA
Previous instrumental vaginal delivery, n (%)	NA	NA	269 (11.0)	NA	NA	NA
Previous caesarean birth, n (%)	NA	NA	286 (11.9)	NA	NA	NA
Previous planned caesarean birth, n (% of all sections)	NA	NA	110 (38.5)	NA	NA	NA

Abbreviations: NA = Not applicable to this group, SD = standard deviation

**Table 2:** The comparison of posttraumatic stress symptoms, fear of childbirth, and depression between women and partners

Questionnaire	Women	Partners
IES score, mean ± SD (range)	17.8 ± 16.3 (0–83)*	14.3 ± 14.5 (0–71)*
IES ≥ 24, %	32.2*	24.1*
IES ≥ 33, %	20.2*	13.4*
W-DEQ-A score, mean ± SD (range)	63.1 ± 22.7 (4-156)*	37.7 ± 17.4 (0-124)*
W-DEQ-A ≥ 85, %	16.6*	0.8*
W-DEQ-A ≥ 100, %	5.9*	0.3*
VAS, mean ± SD (range)	42.0 ± 25.7 (0-100)*	24.6 ± 19.5 (0-100)*
VAS ≥ 50, %	40.7*	15.3*
EPDS score mean ± SD (range)	6.0 ± 4.1 (1–28)*	3.5 ± 3.3 (0–27)*
EPDS ≥ 10, %	18.4*	5.7*
EPDS ≥ 10, %	7.6*	1.8*

Abbreviations: IES = Impact of Event Scale, W-DEQ-A = Wijma Delivery Expectancy Questionnaire, VAS = Visual analogue scale

*p < 0.001 in comparisons between women and their partners (made with Mann-Whitney u-tests for abnormally distributed variables, independent samples t-tests for normally/normally alike distributed variables, and Chi-Square tests for categorical variables)

Table 3 shows the comparison of symptoms suggesting PTSS, FOC, depression, and HRQoL between those without and those with previous children. Nulliparous women as well as partners without previous children were more scared of childbirth than those with previous children when measured with means of W-DEQ-A and VAS. Women’s or partners’ PTSS, depression symptoms, or HRQoL did not differ between those with and without previous children.

**Table 3:** The comparison of posttraumatic stress symptoms, fear of childbirth, and depression between those without and those with previous children

Questionnaire	Nulliparous women	Parous women	Partners without previous children	Partners with previous children
IES score, mean ± SD (N)	17.7 ± 13.0 (1807)	18.0 ± 15.8 (1445)	13.7 ± 13.7 (810)	14.5 ± 14.8 (1025)
IES ≥ 24, N (% of all answers)	596 (17.5)	475 (14.6)	177 (9.6)	252 (13.7)
IES ≥ 33	374 (11.5)	282 (8.7)	95 (5.2)	143 (7.8)
W-DEQ-A score, mean ± SD (N)	67.3 ± 22.2 (2003)*	57.7 ± 22.1 (1564)*	40.2 ± 16.9 (919)*	34.4 ± 17.0 (1201)*
W-DEQ-A ≥ 85, N (% of all answers)	417 (11.7)*	175 (4.9)*	6 (0.3)	10 (0.5)
W-DEQ-A ≥ 100, N (% of all answers)	150 (4.2)*	60 (1.7)*	2 (0.1)	3 (0.1)
VAS, mean ± SD (N)	45.9 ± 24.9 (2138)*	36.8 ± 25.9 (1663)*	26.7 ± 19.7 (987)*	20.6 ± 17.9 (1285)*
VAS ≥ 50, N (% of all answers)	981 (25.8)*	564 (14.8)*	175 (7.7)*	131 (5.8)*
EPDS score mean ± SD (N)	5.9 ± 4.1(2114)	6.10 ± 4.2 (1644)	3.3 ± 3.1 (972)	3.6 ± 3.5 (1280)
EPDS ≥ 10	391 (10.4)	300 (8.0)	46 (2.0)	84 (3.7)
EPDS ≥ 10	149 (4.0)	139 (3.7)	13 (0.6)	25 (1.1)
15D score mean ± SD (N)	0.928 ± 0.061 (2087)	0.924 ± 0.060 (1612)	0.968 ± 0.047 (967)	0.966 ± 0.044 (1256)

Abbreviations: IES = Impact of Event Scale, W-DEQ-A = Wijma Delivery Expectancy Questionnaire, VAS = Visual analogue scale

*p < 0.001 in comparisons between nulliparous and parous women or between partners without and with previous children (made with Mann-Whitney u-tests for abnormally distributed variables, independent samples t-tests for normally/normally alike distributed variables, and Chi-Square tests for categorical variables)

In the IES, we asked about life events that the pregnant women and their partners found especially stressful. Only 9.8% of women and 34.9% of partners did not report such an event in life. For the women, the three most common events were the death of a close person (mentioned by 18.5%), personal health issues (18.1%), and sickness of a close person (12.4%). Respectively, in partners the three most common events were the death of a close person (14.5%), unemployment/financial difficulties (10.7%), and sickness of a close person (8.7%).

As far as PTSS is concerned, 9.7% of the couples had IES ≥ 24 and 3.4% had IES ≥ 33. However, symptoms suggesting FOC and depression rarely occurred simultaneously in both partners when measured with W-DEQ-A and EPDS, as seen in Table 4. If a partner reported symptoms associated with PTSS, FOC, or depression, the pregnant woman more often reported symptoms associated with the same disorder than vice versa.

**Table 4:** Couples’ mutual traumatic symptoms, fear of childbirth, and depression

The cut-off values	Both in a couple exceeding the cut-off values, %	When woman exceeded the cut-off value, also her partner exceeded the same cut-off value, %	When partner exceeded the cut-off value, also the woman exceeded the same cut-off value, %
IES score ≥ 24	9.7	19.6	35.5
IES score ≥ 33	3.4	10.9	22.3
W-DEQ-A ≥ 85	0.3	1.4	36.4
W-DEQ-A ≥ 100	0.04	0.5	14.3
VAS ≥ 50 mm	7.6	18.9	50.2
EPDS score ≥ 10	2.0	8.5	20.5
EPDS score ≥ 13	0.4	3.8	20.4

Abbreviations: IES = Impact of Event Scale, W-DEQ-A = Wijma Delivery Expectancy Questionnaire, VAS = Visual analogue scale

**Fig. 1:**
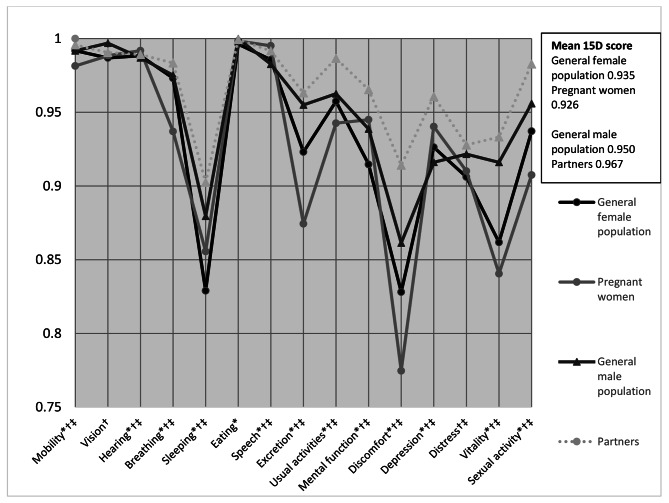
The mean 15D profile of the pregnant women and their partners compared with each other and those of the age-and gender-standardized general population * p < 0.05 in independent samples t-test between pregnant women and age-standardized general female population. † p < 0.05 in independent samples t-test between partners and age-standardized general male population. ‡ p < 0.05 in independent samples t-test between pregnant women and their partners.

Pregnant women’s and their partners’ mean 15D profiles are shown in Fig. 1 and compared with each other and with those of the age- and gender-standardized general population [38]. Pregnant women’s mean 15D score 0.926 (SD 0.061, range 0.55-1.00) differed statistically significantly and clinically importantly from their partners’ mean score 0.967 (SD 0.045, range 0.106-1.000) (p < 0.001). Women’s mean dimension level values, except for vision (p = 0.10) and eating (p = 0.079), differed also statistically significantly from those of their partners (hearing p = 0.036 and speech p = 0.04, all others p < 0.001), and all their mean dimension level values were lower than those of partners’, except speech (communication) and hearing. In pregnant women the mean 15D score and the mean dimension level values, except for seeing (p = 0.09) and distress, (p = 0.09) differed statistically significantly from those of the age-standardized general female population [38] (p < 0.001). In partners, the mean 15D score and the mean dimension level values, except for eating, (p = 0.12) differed statistically significantly from those of the age-standardized general male population [38] (hearing p = 0.04 and distress p = 0.01, and all others p < 0.001).

## Discussion

In this large, unselected community study population, every third pregnant woman seemed to have at least partial PTSD or some of the PTSS (IES score ≥ 24) and in every fifth pregnant woman PTSD was likely present (IES score ≥ 33). To our knowledge, partners’, or couples’ prevalence of PTSS/PTSD during pregnancy and possible differences in PTSS between women and partners without and with previous children have not been studied before. Although partners had less symptoms suggesting PTSS/PTSD than pregnant women, these symptoms were still common as almost every fourth partner had at least some PTSS and in every eighth PTSD was likely present. In 10% of couples both had an IES score ≥ 24 and in 3% score ≥ 33, which indicates that in many pregnant couples there is a burden complicating everyday life and preparing for parenthood. Symptoms suggesting PTSS and depression were as common both in women and partners with and without previous children, but FOC was more common in those without previous children. Symptoms associated with FOC and depression were common in women but uncommon in partners, and thus they rarely occurred simultaneously in couples. However, women whose partners reported PTSS, FOC, or depressive symptoms, often reported the same symptoms. Consequently, special attention should be paid to a pregnant woman whose partner suffers from any of these symptoms.

Our results suggest a high prevalence of PTSS/PTSD in a community sample of pregnant women, which raises concern as in a systematic review and meta-analysis the mean prevalence of prenatal PTSD was 18–19% in high-risk women and only 3% in community samples [21, 23]. On the other hand, these studies found high variation in the prevalence due to different ways of measuring, and in pregnant women’s community samples the prevalence of PTSD varied between 0 and 21% [21, 23].

FOC and depressive symptoms were common in women, and in line with earlier studies using the same measures and same cut-off values [4–6, 11, 32]. This study confirmed that nulliparous women had more FOC than parous women [32]. The same was found in partners both in our study as well as previously [13]. There are contradictory results about the effect of parity to depressive symptoms as in some studies multiparous women were those at increased risk of developing prenatal depression, while in some studies nulliparous were those at increased risk, and in others no association between parity and prenatal depression was found [8, 39]. However, regarding both the women and their partners in our study, depressive symptoms were equally common in those without and with previous children. During pregnancy, women were more scared of childbirth, had more depressive symptoms, and had worse HRQoL than partners as reported also previously [7, 9, 12]. In this study, partners’ FOC prevalence measured with W-DEQ-A was clearly lower than in earlier few studies from Sweden (11–14%) [13, 17], which, however, used different measures and different cut-off values. Yet, FOC measured with VAS was more in line with their results [13, 17]. Our results about FOC measured with W-DEQ-A are more in line with an earlier Finnish study, where none of the 250 partners had W-DEQ-A ≥ 100 and only one scored W-DEQ-A ≥ 85 [12]. In meta-analyses including studies using EPDS, the prevalence of partners’ prenatal mild depressive symptoms was 1-12% [4, 35] and clinically relevant depressive symptoms 2-5% [4, 7, 35]. Our results are quite in line with these results.

The couples rarely shared mutual FOC (severe 0.3% and phobic 0.04%) when measured with W-DEQ-A, which was quite the same result as in a Swedish study (0.7%) [16]. We offer two possible explanations for this. First, it is possible that couples in which both suffer from severe FOC do not choose to have children, but there is no research about the matter. Second, because partners reported FOC and depressive symptoms quite seldom, only in a few couples did both parties share these same symptoms. The latter explanation is supported by our finding that the prevalence of symptoms associated with FOC in partners and in couples were much higher with VAS than with W-DEQ-A. However, paternal prenatal depression might be a considerable risk for maternal prenatal depression in ours as well as in other studies [4, 7, 8]. In fact, when a pregnant woman’s partner reported depressive symptoms, then in every fifth case also the woman experienced depressive symptoms, similarly to a New-Zealand study [7]. Moreover, if a pregnant woman’s partner reported PTSS or FOC, also a notable proportion of pregnant women reported the same condition of the same magnitude. A possible explanation for this might be lowered support from a pregnant woman’s partner, as lack of support and dissatisfaction with the partnership have been very strong predictors of severe FOC [14], and as low social support has strong associations with depression and other mental health problems in women [40].

It is important to recognize and treat FOC during pregnancy as it may overshadow the entire pregnancy, complicate childbirth, and lead to a negative childbirth experience, difficulties in the mother-infant relationship, PTSD after childbirth, and increase the risk of future voluntary infertility [11, 15, 17–20]. Treatment of FOC in women seems to at least reduce the number of caesarean sections  [15, 41] and improve the childbirth experience [15], and it may also help fearful partners get a more positive childbirth experience [17]. This study confirmed the earlier findings using the VAS in screening for phobic FOC in women: even though VAS is not as accurate as W-DEQ A, its simplicity promotes high compliance, and it is very sensitive [32].

Pregnant women’s HRQoL was at a similar level with previous studies [42]. According to an earlier study, the HRQoL of pregnant women was worse than that of the general female population of the same age [26]. In our study, HRQoL, measured mostly during the second trimester of pregnancy, was lower especially because of more difficulties with mobility, breathing, excretion, usual activities, discomfort, vitality, and sexual activity, even though pregnancy-induced physiological changes should not have yet had a major impact on them. However, the difference in the mean 15D score between women and that of the age-standardized general female population was statistically significant due to large sample sizes, but probably not clinically important [37]. Women’s physical and emotional self-rated health had earlier been affected negatively as pregnancy progresses, while partners’ self-rated health had stayed stable throughout the pregnancy [27]. The results showed partners’ HRQoL as statistically and clinically significantly [37] better than that of pregnant women’s and that of the age-standardized general male population. Although an earlier study had found previous parity affecting negatively to physical and/or emotional self-rated health (both measured with one question) in women and men [27], neither our study nor another previous study [26] found any differences between the nulliparous and parous.

Prenatal depression, anxiety or stress might have a significant negative influence on pregnancy, birth outcome, the time after pregnancy, family, and child developmental outcome both in women and their partners [1, 2, 4, 5, 7, 8, 42]. Nowadays, poor mental health is often unrecognized [1–3] even though routine pregnancy visits provide several opportunities to identify and support women with mental health problems and social adversity [3]. In an Australian study only around half of pregnant participants were asked about depressive symptoms, anxiousness, or worries during routine visits [3]. Furthermore, even though patients reported poor self-rated mental health during pregnancy, only 19% of them consulted a healthcare professional for mental health problems [2]. There seems to be an enormous gap between the need for help and the recognition of those in need of help. Further, we should not leave a pregnant woman’s partner’s mental state without attention because of its possible effect on the pregnant woman’s mental health, possible consequences on the partner’s own future mental health, and on the future child [4, 7]. Our results confirm the earlier noticed need both for screening of FOC, depression, and PTSS/PTSD in maternity care including the partner and referring to adequate treatment to prevent later consequences to the whole family [1–3, 5, 7, 8, 15, 17, 22, 23].

Our study had some limitations that should be considered when generalizing the findings of the study. First, as participation in the study was voluntary, we cannot exclude the possibility that participating was open for bias. However, our study sample can be assumed to be representative because its background characteristics equate with the general Finnish pregnant population [10, 43]. Second, although couples were asked to return their surveys separately, it was not controlled that the couple did not complete the surveys together. Third, some immigrants were probably left out of the study because of poor skills in either Finnish or Swedish language. Fourth, the time window for completing the surveys was wide during pregnancy. Fifth, questionnaires and self-reporting of symptoms do not alone justify diagnosis, as clinical evaluation is always needed to diagnose the disorders searched in our study. Finally, IES-R does not require exposure to/threat of death/ sexual violence/injury but the person answering to IES-R can determine whether they have had any kind of traumatizing event, which can influence the prevalence. Moreover, there were no previous studies using IES to measure prenatal PTSS or PTSD.

The greatest strengths of this unusually large questionnaire study in the capital area of Finland, where every third Finnish child is born, were that it examined not only an unselected population of pregnant women and their partners, but analyzed the data also as couples. To our knowledge, this study is the first to show that paternal prenatal PTSS and FOC might carry a considerable risk of similar symptoms in pregnant women. The results help us understand how common poor mental health is among pregnant couples and to focus our support, care, and resources on them.

## Conclusions

During pregnancy, PTSS and probable PTSD were surprisingly common in both women and partners, and shared in several couples. Ordinary hardships of life, such as death or sickness of a close person, seemed to pose a considerable stress factor for families expecting a child. Pregnant women seemed to experience FOC and depressive symptoms to the same extent as shown in previous studies and their HRQoL was at a similar level with previous studies. In turn, their partners reported much less FOC and depressive symptoms, and thus FOC and depressive symptoms rarely occurred simultaneously in both partners. The HRQoL of the partners was clinically significantly better than that of the age-standardized general male population, while the differences in HRQoL between pregnant women and that of the age-standardized general female population were probably clinically unimportant. Nulliparous women and partners without previous children seem to experience FOC more often than those with previous children, but there was no difference in PTSS, depression, or HRQoL. Special attention should be paid to pregnant women whose partners feel mentally ill, because there was a great chance that the woman experienced the same symptoms without getting support from her partner. According to previous studies, both PTSS and FOC as well as depression might have serious consequences if not treated, and yet they are poorly detected. Considering all previous and our results, we suggest routine prenatal screening of PTSD, FOC, and depression in primary maternity care, including the partner, with related referral to adequate treatment.

## Data Availability

The datasets generated and/or analyzed during the current study are not publicly available due to limitations of ethical approval involving the patient data and anonymity but are available from the corresponding author on reasonable request.

## List of Abbreviations

15D: 15D instrument
EPDS: Edinburgh Postnatal Depression Scale
FOC: Fear of childbirth
HRQoL: Health-related quality of life
IES-R: Impact of Event Scale revised version
IES: Impact of Event Scale
PTSD: Posttraumatic stress disorder
PTSS: Posttraumatic stress symptoms
SD: Standard deviation
VAS: Fear of childbirth Visual Analogue Scale
W-DEQ-A: Wijma Delivery Expectancy Questionnaire
